# Racial and Ethnic Differences in Dietary Intake and Metabolic Profiles Among Adults with Heart Failure, Type 2 Diabetes, and Overweight or Obesity: A Secondary Analysis of the Pro-HEART Trial

**DOI:** 10.3390/nu18162737

**Published:** 2026-08-21

**Authors:** Lorraine S. Evangelista, Angelina P. Nguyen, Jennifer Kawi, Rebecca Meraz, Kelly L. Wierenga, Alona D. Angosta, Michele A. Hamilton, Gregg C. Fonarow

**Affiliations:** 1 Sue & Bill Gross School of Nursing, University of California Irvine, Irvine, CA 92697, USA; 2 Louise Herrington School of Nursing, Baylor University, Dallas, TX 75246, USA; angelina_nguyen@baylor.edu (A.P.N.); rebecca_meraz@baylor.edu (R.M.);; 3 Cizik School of Nursing, The University of Texas Health Science Center at Houston, Houston, TX 77030, USA; jennifer.kawi@uth.tmc.edu; 4 Smidt Heart Institute, Cedars-Sinai Medical Center, Los Angeles, CA 90048, USA; 5 Ahmanson-UCLA Cardiomyopathy Center, Division of Cardiology, David Geffen School of Medicine, University of California, Los Angeles, Los Angeles, CA 90095, USA

**Keywords:** heart failure, dietary intake, race and ethnicity, overweight and obesity, laboratory biomarkers, nutrition assessment, cardiovascular health, secondary analysis

## Abstract

**Background:** Racial and ethnic disparities in heart failure (HF) outcomes are well documented, but little is known about dietary intake and metabolic profiles among individuals with chronic HF and overweight or obesity. This exploratory secondary analysis of baseline data from the Pro-HEART randomized trial compared dietary intake and laboratory biomarkers across racial and ethnic groups. **Methods:** This secondary analysis used baseline data from the Pro-HEART randomized clinical trial (ClinicalTrials.gov identifier: NCT01423266). Baseline data from 92 adults with chronic HF, type 2 diabetes mellitus (DM), and overweight or obesity were analyzed. Participants self-identified as White (*n* = 49), Asian/Other (*n* = 17), Hispanic (*n* = 14), or Black (*n* = 12). Dietary intake was assessed using 3-day food records, which were analyzed with the Nutrition Data System for Research. Selected biomarkers included lipid measures, fasting glucose, hemoglobin A1c, albumin, total protein, and creatinine. Group differences were evaluated using one-way analysis of variance and chi-square or Fisher’s exact tests, as appropriate. Because multiple outcomes were examined without a prespecified primary endpoint, *p*-values were not adjusted for multiplicity and are reported as descriptive. **Results:** Participants had a mean age of 58.5 ± 9.7 years, were predominantly male (69.6%), married (64.0%), and had New York Heart Association Class II HF (75.0%). Mean age differed across groups (omnibus *p* = 0.046); no pairwise contrast remained significant after Bonferroni adjustment. No statistically significant between-group differences were identified in the available data for body mass index, waist circumference, left ventricular ejection fraction, dietary intake, or laboratory biomarkers (all *p* > 0.05); however, the small subgroup sizes and substantial missing dietary data limited the precision of these comparisons. **Conclusions:** In this exploratory cohort of adults with chronic HF, type 2 DM, and overweight or obesity, no statistically significant racial or ethnic differences were identified in the dietary and laboratory measures examined. However, the small subgroup sizes and substantial missing dietary data limit the precision of these findings, and smaller or clinically meaningful differences cannot be excluded. Larger studies with more complete dietary data are needed to better characterize potential racial and ethnic differences in nutrition and metabolic health in this population.

## 1. Introduction

Heart failure (HF) affects an estimated 64 million people worldwide and continues to be a leading cause of hospitalization and death [1]. Although treatment has improved substantially over the past two decades, many people living with HF continue to experience persistent symptoms, limited physical function, and repeated hospitalizations [2,3]. Nutrition has become an important part of HF management because it offers another way to support health alongside medications and device therapy.

Diet in HF involves more than salt and fluid restriction. Researchers are also paying closer attention to protein intake, overall food intake, and micronutrient adequacy because these factors may affect inflammation, metabolic health, muscle preservation, and physical function [4,5]. Dietary approaches such as the Mediterranean and Dietary Approaches to Stop Hypertension diets have been associated with better cardiovascular health. At the same time, “Food is Medicine” programs emphasize the role of healthy foods in the routine care of chronic disease [5]. Even when calorie intake is adequate or high, some people with HF may consume diets that are high in energy but low in important nutrients, placing them at risk for poor nutritional and metabolic health [1,5].

Traditionally, cachexia and low body weight in people with advanced HF have been associated with malnutrition and nutritional deficiencies. However, nutritional risk is also common among people with overweight or obesity, who now represent a substantial proportion of the HF population [4,6]. In our recent analysis of participants in the Pro-HEART trial, many individuals reported high intakes of saturated fat and sodium while failing to meet recommendations for several important micronutrients, including vitamin D, calcium, magnesium, and potassium [6]. These findings showed that a higher body weight does not necessarily indicate adequate nutritional status. The quality and nutrient content of the diet also matter.

Food choices do not occur in isolation. Food choices are influenced by cultural practices, food availability, economic conditions, access to health care, and other social factors that may also contribute to racial and ethnic differences in cardiovascular risk [5,7]. Although disparities in cardiovascular outcomes are well documented, less is known about whether dietary intake and metabolic profiles differ across racial and ethnic groups among people living with HF. Examining these differences may help guide future nutrition interventions that are more responsive to the needs and circumstances of diverse populations.

Previous population-based studies have reported racial and ethnic differences in diet quality and cardiometabolic risk. Findings from NHANES, the Multi-Ethnic Study of Atherosclerosis, and the Jackson Heart Study suggest that dietary intake and cardiovascular risk factors vary across racial and ethnic groups [8,9,10]. These findings provided a rationale for examining whether similar differences were present among adults with HF, type 2 diabetes mellitus (DM), and overweight or obesity. Dietary differences may be particularly relevant in this population because adults with HF, type 2 DM, and overweight or obesity often receive multiple, sometimes competing, dietary recommendations related to sodium restriction, glycemic control, weight management, and overall cardiovascular risk reduction. Cultural food practices, food access, socioeconomic resources, and prior nutrition counseling may also influence how these recommendations are understood and followed. These factors could contribute to differences in dietary intake across racial and ethnic groups even among individuals with similar clinical conditions. However, because these differences have been less frequently studied in HF populations, the present analysis was exploratory rather than hypothesis-driven. The Pro-HEART trial provided an opportunity to examine these questions in a well-characterized cohort. Earlier findings showed that dietary intervention improved selected cardiometabolic outcomes [11], while a later analysis identified poor dietary quality and micronutrient inadequacy despite excess body weight [6]. The present analysis builds on that work by examining whether dietary intake and laboratory biomarkers differed across racial and ethnic groups.

## 2. Materials and Methods

### 2.1. Study Design and Participants

This study was a cross-sectional exploratory analysis of baseline data collected from participants enrolled in the Pro-HEART randomized controlled trial (ClinicalTrials.gov identifier: NCT01423266). The parent study evaluated the effects of a high-protein dietary intervention in adults living with chronic HF, type 2 DM, and overweight or obesity. Previous reports have detailed the design and primary findings of the Pro-HEART trial [11].

Adults with stable chronic HF were recruited from outpatient cardiology clinics affiliated with a large academic medical center in Southern California. Eligibility criteria for the parent trial included stable chronic HF, type 2 DM, and overweight or obesity, as it was designed to evaluate a dietary intervention in adults with both HF and type 2 DM. Additional inclusion and exclusion criteria, including the body mass index threshold, have been reported previously [8].

All participants provided written informed consent before enrollment. The study protocol was approved by the Institutional Review Boards of the University of California, Irvine (HS 2010-7972; approved 12 March 2012), and the University of California, Los Angeles (IRB#10-000471-AM-00009, approved 28 November 2011). All study procedures complied with the ethical principles outlined in the Declaration of Helsinki.

### 2.2. Race and Ethnicity

Race and ethnicity were self-reported using categories available in the parent Pro-HEART dataset. Because of very small cell sizes, participants originally coded as Asian, Native Hawaiian, Mixed, or Other could not be analyzed separately and were combined into a single heterogeneous analytic category, labeled “Asian/Other” for the present analysis. This grouping was used solely to enable exploratory statistical comparisons and should not be interpreted as implying that these racial and ethnic identities are equivalent.

For a small number of participants with incomplete original coding, the enrollment record was reviewed to assign a single analytic category based on documented self-identification. Comparisons of dietary intake and laboratory biomarkers were performed across the four analytic groups. Race and ethnicity are social rather than biological constructs. These categories were used to describe participants’ self-identified racial and ethnic backgrounds and were not interpreted as markers of inherent biological differences. Because key social determinants of health, including food security, income, neighborhood food access, and medication access, were not measured, the analyses cannot determine the social or structural mechanisms that may underlie racial and ethnic differences in HF outcomes.

### 2.3. Dietary Assessment

Dietary intake was assessed using three-day food records consisting of two weekdays and one weekend day. Participants received standardized instructions from trained study personnel on recording all foods and beverages consumed, including portion sizes and methods of preparation.

Completed food records were analyzed using the Nutrition Data System for Research (Nutrition Coordinating Center, University of Minnesota, Minneapolis, MN, USA) [12]. Average daily energy and macronutrient intakes were calculated from the three-day food records and used in the present analysis. Sodium and selected micronutrients were available from the dietary records but were not included in this secondary analysis. The present analysis was intentionally limited to energy and macronutrient measures to maintain a focused, parsimonious set of dietary comparisons, given the small racial and ethnic subgroup sizes and substantial missing dietary data. Micronutrient intake had been examined previously in the Pro-HEART cohort [6] and was therefore outside the scope of the present analysis.

All dietary records were collected at baseline, before randomization and before participants received any study-related dietary counseling or intervention. Energy-reporting plausibility was not formally assessed using the Goldberg cutoff or other energy-intake plausibility criteria, and dietary records were not excluded on the basis of reported energy intake.

### 2.4. Metabolic Biomarkers

Fasting blood samples were obtained at baseline in accordance with standardized study procedures. Laboratory measures included traditional lipid and glycemic biomarkers, including total cholesterol, low-density lipoprotein cholesterol, high-density lipoprotein cholesterol, triglycerides, fasting glucose, and hemoglobin A1c, as well as albumin, total protein, and creatinine. These biomarkers were selected because of their relevance to cardiometabolic health and nutritional status in adults living with HF.

### 2.5. Statistical Analysis

Descriptive statistics were used to summarize participant characteristics, dietary intake, and metabolic biomarkers. Continuous variables are presented as means and standard deviations; categorical variables are summarized as frequencies and percentages.

Differences among racial and ethnic groups in continuous variables were evaluated using one-way analysis of variance (ANOVA), with Bonferroni-adjusted post hoc comparisons when appropriate. Categorical variables were compared using the chi-square or Fisher’s exact test, as appropriate. Statistical significance was defined as a two-sided *p* < 0.05.

Given the fixed sample size (N = 92), the minimum detectable effect size for a four-group omnibus ANOVA at α = 0.05 and 80% power was approximately Cohen’s f = 0.35 (η^2^ ≈ 0.11), indicating that the study was primarily powered to detect moderate-to-large between-group differences.

Because this was an exploratory analysis without a prespecified primary endpoint, *p*-values were not adjusted for multiple comparisons and are presented descriptively. Eta-squared (η^2^) effect sizes and 95% confidence intervals were estimated using the fixed-effect one-way ANOVA effect-size procedure model in IBM SPSS Statistics, Version 27 [13] and are reported for continuous outcomes. For outcomes requiring Welch’s ANOVA because of unequal variances, the Welch test was used for statistical inference. At the same time, η^2^ and its 95% confidence interval were retained as descriptive omnibus effect-size estimates from the conventional ANOVA model. Analyses were based on available complete cases for each outcome, and missing values were not imputed. The number of participants contributing to each analysis is reported. Reasons for individual missing laboratory or dietary values were not systematically documented in the dataset used for this secondary analysis. Because age differed across racial and ethnic groups at baseline, age-adjusted sensitivity analyses were conducted using general linear models with race/ethnicity as the fixed factor and age as a covariate. All analyses were performed using IBM Statistical Package for the Social Sciences, Version 27.0 (IBM Corp., Armonk, NY, USA) [13].

## 3. Results

### 3.1. Participant Characteristics

The study included 92 adults with chronic HF and overweight or obesity, including 49 White (53.3%), 17 Asian/Other (18.5%), 14 Hispanic (15.2%), and 12 Black (13.0%) participants. The mean age of the cohort was 58.5 ± 9.7 years. Most participants were male (69.6%), married (64.0%), and had a college education or higher (77.2%). Approximately three-quarters of the cohort had New York Heart Association (NYHA) Class II HF, with the remainder classified as NYHA Class III.

Except for age, no statistically significant between-group differences were detected in the baseline demographic or clinical characteristics. Mean age differed across groups (omnibus ANOVA, *p* = 0.046); Hispanic participants were the youngest (52.6 ± 11.4 years), and White participants were the oldest (60.3 ± 9.8 years), although no pairwise contrast remained significant after Bonferroni adjustment. No significant differences were observed for educational attainment, body mass index, left ventricular ejection fraction, gender, marital status, NYHA functional class, or waist circumference (Table 1). The amount of missing data varied by outcome and was greatest for the dietary variables, with contributing sample sizes ranging from 29 to 39 participants. Among laboratory measures, fasting glucose was available for 76 participants, albumin for 50, and creatinine for 91; the remaining biomarkers were available for all 92 participants. Contributing sample sizes by racial and ethnic groups are reported in Table 2 and Table 3. For several dietary measures, subgroup cell sizes were extremely small, limiting the stability and interpretability of between-group comparisons.

### 3.2. Dietary Intake by Race and Ethnicity

No statistically significant between-group differences were detected in daily energy intake or the dietary variables examined (Table 2). However, these comparisons should be interpreted very cautiously because the dietary analyses included only 29–39 participants overall, and several racial and ethnic subgroup cells contained as few as two to three participants. Mean daily energy intake ranged from approximately 1426 to 1946 kcal/day across groups, but the small and unequal subgroup sizes substantially limit the interpretation of apparent numerical differences. Carbohydrates accounted for approximately 39% to 49% of total energy intake, protein contributed 22% to 31%, and fat contributed approximately 29% to 37%. No statistically significant differences in macronutrient distribution were identified across groups (all *p* > 0.05).

**Table 2:** Baseline dietary intake by racial and ethnic group.

Dietary Variable	Black	White	Asian/Other	Hispanic	Omnibus *p*	η^2^ (95% CI)
Energy intake, kcal/day	1502.5 ± 708.1 (4)	1426.2 ± 634.8 (21)	1945.9 ± 797.0 (7)	1578.6 ± 752.8 (7)	0.406	0.079 (0.000–0.218)
Total carbohydrate, g/day	175.6 ± 90.6 (4)	141.6 ± 63.3 (20)	206.4 ± 107.0 (7)	187.2 ± 118.5 (7)	0.325	0.096 (0.000–0.245)
Dietary fiber, g/day	15.8 ± 13.0 (3)	17.4 ± 11.0 (20)	20.6 ± 6.0 (7)	21.9 ± 16.8 (7)	0.770	0.033 (0.000–0.132)
Energy from carbohydrates, %	39.2 ± 21.2 (3)	39.0 ± 8.8 (15)	49.3 ± 8.3 (6)	44.5 ± 7.0 (5)	0.191	0.170 (0.000–0.353)
Total protein, g/day	88.2 ± 19.0 (4)	74.4 ± 30.1 (20)	78.6 ± 20.8 (7)	77.4 ± 32.2 (7)	0.846	0.023 (0.000–0.102)
Animal protein, g/day	59.6 ± 2.8 (4)	56.7 ± 26.8 (20)	52.9 ± 12.8 (7)	53.4 ± 20.0 (7)	0.950	0.010 (0.000–0.038)
Vegetable protein, g/day	28.6 ± 16.5 (4)	17.7 ± 9.1 (20)	25.7 ± 9.8 (7)	24.0 ± 12.5 (7)	0.148	0.144 (0.000–0.307)
† Energy from protein, %	30.7 ± 16.9 (3)	23.7 ± 5.2 (15)	22.2 ± 7.2 (6)	25.0 ± 3.7 (5)	0.796	0.109 (0.000–0.278)
Total fat, g/day	51.2 ± 38.5 (4)	62.5 ± 35.6 (21)	60.8 ± 33.2 (7)	58.3 ± 25.8 (7)	0.940	0.011 (0.000–0.047)
Saturated fat, g/day	13.5 ± 9.6 (3)	19.2 ± 11.0 (20)	19.7 ± 10.0 (6)	19.0 ± 8.0 (6)	0.828	0.028 (0.000–0.117)
Monounsaturated fat, g/day	14.4 ± 12.5 (2)	24.5 ± 15.0 (16)	25.0 ± 12.6 (6)	21.4 ± 10.0 (6)	0.755	0.044 (0.000–0.164)
Polyunsaturated fat, g/day	13.1 ± 14.1 (2)	16.0 ± 13.9 (16)	16.0 ± 8.6 (6)	15.5 ± 8.7 (6)	0.991	0.004 ‡
Energy from fat, %	29.0 ± 11.9 (3)	36.7 ± 7.3 (15)	28.5 ± 9.0 (6)	30.7 ± 7.5 (5)	0.134	0.197 (0.000–0.381)

Footnote: Values are presented as mean ± standard deviation (contributing *n*). Dietary records were collected at baseline before randomization and before study-related dietary counseling or intervention. Omnibus *p*-values are from one-way analysis of variance, except † Welch’s robust test was used because the homogeneity-of-variance assumption was not met. Eta squared (η^2^) and 95% confidence intervals were estimated from the fixed-effect one-way analysis of variance model and are presented as measures of effect magnitude; for outcomes analyzed using Welch’s test, Welch’s *p*-value is reported while η^2^ is provided as a descriptive effect-size estimate. *p*-values are descriptive and were not adjusted for multiplicity. Missing values were not imputed. ‡ For polyunsaturated fat, the SPSS effect-size procedure returned a boundary confidence interval of 0.000–0.000; because this interval did not provide a meaningful estimate of precision, it is reported as not estimable.

### 3.3. Laboratory Biomarkers

No statistically significant between-group differences were detected in the traditional lipid and glycemic biomarkers or other laboratory measures examined (Table 3). Mean hemoglobin A1c ranged from 6.94% to 7.38%, and mean fasting glucose ranged from 120.7 to 144.0 mg/dL. Age-adjusted sensitivity analyses did not materially change the findings; no statistically significant racial or ethnic group differences were detected in the dietary or laboratory outcomes examined.

**Table 3:** Baseline laboratory biomarkers by racial and ethnic group.

Biomarker	Black	White	Asian/Other	Hispanic	Omnibus *p*	η^2^ (95% CI)
Total cholesterol, mg/dL	157.4 ± 44.1 (12)	163.7 ± 42.0 (49)	163.4 ± 38.5 (17)	153.6 ± 40.9 (14)	0.853	0.009 (0.000–0.043)
Low-density lipoprotein cholesterol, mg/dL	86.8 ± 37.1 (12)	93.8 ± 31.2 (49)	89.7 ± 36.0 (17)	99.1 ± 37.3 (14)	0.791	0.012 (0.000–0.054)
† High-density lipoprotein cholesterol, mg/dL	36.3 ± 7.5 (12)	42.8 ± 13.5 (49)	40.6 ± 7.4 (17)	39.1 ± 9.7 (14)	0.193	0.040 (0.000–0.117)
Triglycerides, mg/dL	139.2 ± 61.5 (12)	152.8 ± 55.4 (49)	184.8 ± 81.8 (17)	165.5 ± 46.8 (14)	0.179	0.054 (0.000–0.141)
Fasting glucose, mg/dL	137.9 ± 57.3 (10)	120.7 ± 57.2 (39)	134.0 ± 35.5 (13)	144.0 ± 64.2 (14)	0.528	0.030 (0.000–0.105)
Hemoglobin A1c, %	7.38 ± 1.54 (12)	6.94 ± 1.43 (49)	7.26 ± 1.01 (17)	7.38 ± 2.08 (14)	0.649	0.018 (0.000–0.073)
Albumin, g/dL	4.20 ± 0.44 (8)	4.26 ± 0.25 (24)	4.27 ± 0.38 (10)	4.18 ± 0.47 (8)	0.907	0.012 (0.000–0.056)
† Total protein, g/dL	6.89 ± 0.46 (12)	6.72 ± 0.22 (49)	6.78 ± 0.16 (17)	6.65 ± 0.67 (14)	0.408	0.037 (0.000–0.112)
Creatinine, mg/dL	1.13 ± 0.25 (12)	1.11 ± 0.30 (49)	1.09 ± 0.31 (16)	1.06 ± 0.35 (14)	0.918	0.006 (0.000–0.028)

Footnote. Values are presented as mean ± standard deviation (contributing *n*). Omnibus *p*-values are from one-way analysis of variance, except † Welch’s robust test was used because the homogeneity-of-variance assumption was not met. Eta squared (η^2^) and 95% confidence intervals were estimated from the fixed-effect one-way analysis of variance model and are presented as measures of effect magnitude; for outcomes analyzed using Welch’s test, Welch’s *p*-value is reported while η^2^ is provided as a descriptive effect-size estimate. *p*-values are descriptive and were not adjusted for multiplicity. Missing values were not imputed.

## 4. Discussion

This exploratory secondary analysis assessed dietary intake and selected laboratory biomarkers among Black, White, Asian/Other, and Hispanic adults with chronic HF, type 2 DM, and overweight or obesity. No statistically significant differences were identified across racial and ethnic groups in the dietary or metabolic measures examined, except for mean age. These findings should be interpreted with caution given the small subgroup sizes and the limited precision of the analyses.

Population-based cohorts, including NHANES, the Multi-Ethnic Study of Atherosclerosis, and the Jackson Heart Study, have consistently reported racial and ethnic differences in dietary quality and cardiometabolic risk [8,9,10]. In contrast, participants in the present study were recruited from a single academic medical center, had relatively high educational attainment, and volunteered for a nutrition-focused clinical trial. This recruitment process may have selected individuals who were more motivated or interested in nutrition and who had greater access to health care than the broader HF population. As a result, socioeconomic and behavioral differences across racial and ethnic groups may have been reduced, making between-group differences in diet and cardiometabolic measures more difficult to detect.

These observations are relevant given that there are well-documented racial and ethnic disparities in HF incidence, hospitalization, and mortality. However, these disparities are multifactorial and cannot be explained by a single factor. Diet, clinical risk, health care access, neighborhood factors, and socioeconomic resources all impact HF outcomes [1,5]. Given the limited precision of the present analyses, our findings cannot determine the extent to which baseline dietary intake or laboratory biomarkers contribute to racial and ethnic differences in HF outcomes.

Other factors, including food insecurity, access to health care and medications, financial strain, neighborhood conditions, and differences in the delivery of care, may play a more important role in driving disparities. This interpretation is supported by findings from the REGARDS cohort and the American Heart Association Get With The Guidelines–HF registry, which showed that neighborhood disadvantage was independently associated with worse HF outcomes even after adjustment for clinical risk factors, highlighting the importance of social context in shaping outcomes [14].

Our findings also add to what is known about nutrition among people with HF and overweight or obesity. A higher body weight does not always indicate a positive nutritional status. In prior reporting, we showed that many participants in the Pro-HEART cohort consumed excessive amounts of saturated fat and sodium and did not meet recommendations for several key micronutrients, including vitamin D, calcium, magnesium, and potassium [6].

The relatively high percentage of energy derived from protein was observed in baseline dietary records collected before randomization and before initiation of the dietary intervention. Protein accounted for approximately 22% to 31% of reported energy intake across groups, which is higher than the approximately 15% to 16% typically reported among U.S. adults [15]. Although adequate protein intake is important in HF, particularly for maintaining nutritional status and lean body mass, evidence supporting a single optimal protein intake target for all patients with HF remains limited [16]. The relatively high protein intake in this cohort may partly reflect the characteristics of adults with overweight or obesity who volunteered for a nutrition-focused clinical trial.

Although the present findings are exploratory and limited by small subgroup sizes and missing dietary data, routine nutritional assessment remains an important component of HF care. Assessment should identify inadequate dietary intake, nutritional risk, and barriers to healthy eating and refer to a registered dietitian when a more detailed evaluation or individualized counseling is needed. Nutritional counseling should consider patients’ dietary preferences, financial resources, and access to healthy foods. When appropriate, clinicians should also assess food insecurity and connect patients with Food Is Medicine strategies, such as produce prescriptions, medically tailored meals, or community food resources [5]. Targeted assessment for specific nutrient deficiencies may also be appropriate when dietary intake, clinical findings, or other risk factors suggest it.

Future studies should include larger and more diverse populations, social determinants of health, medication use, and more comprehensive measures of diet quality. Longitudinal studies are also needed to determine whether changes in dietary intake and nutritional status are associated with subsequent hospitalization, functional status, quality of life, and survival. Such studies could help determine whether dietary changes contribute to differences in longer-term HF outcomes across racial and ethnic groups.

The wide distribution of left ventricular ejection fraction in this cohort suggests that participants may have represented more than one HF phenotype. Because this exploratory secondary analysis was not designed or powered to examine differences according to HF phenotype, phenotype-specific analyses were beyond the scope of the present study. Future studies with larger samples should examine whether dietary intake and metabolic profiles differ among patients with HF with reduced ejection fraction, HF with mildly reduced ejection fraction, and HF with preserved ejection fraction.

This study has several strengths. Dietary intake was measured using detailed three-day food records and a standard nutrition database. Participants also completed clinical and laboratory testing using the same study procedures. The study included participants from four racial and ethnic groups, which allowed us to compare dietary intake and laboratory biomarkers across groups.

Despite these strengths, several limitations should be considered when interpreting the findings. First, this was an exploratory secondary analysis of baseline data from a larger randomized trial and cannot establish causal relationships.

Second, the racial and ethnic subgroup sample sizes were small (*n* = 12–49), and the study was primarily powered to detect moderate-to-large between-group effects. Dietary analyses were particularly limited by missing data and very small subgroup sample sizes. Depending on the dietary measure, only 29–39 participants contributed data, with some racial and ethnic subgroup cells containing as few as two to three participants. These small and unequal samples substantially limited precision and the ability to identify potentially meaningful between-group differences. Because the reasons for missing dietary data were not systematically documented, we cannot exclude the possibility that participants with available dietary data differed from those without complete records. If missingness was related to dietary behaviors, disease severity, socioeconomic circumstances, or other participant characteristics, the observed estimates may not accurately represent the full cohort, and between-group differences may have been either attenuated or exaggerated.

Third, dietary intake was assessed using self-reported 3-day food records, which are subject to measurement error and underreporting. The relatively low reported energy intakes in this predominantly male cohort with obesity suggest that underreporting may have occurred. Energy-reporting plausibility was not formally evaluated using the Goldberg cutoff or another objective method, and dietary records were not excluded based on reported energy intake. Because the dietary subgroup samples were already very small, a post hoc analysis excluding participants with potentially implausible energy intakes would have further reduced the available sample and produced even less stable estimates. If underreporting differed across racial and ethnic groups, true between-group differences in dietary intake may have been obscured.

Fourth, traditional lipid and glycemic biomarkers were measured while participants continued their usual clinical care. Medication use, including lipid-lowering and glucose-lowering therapies, was not examined in this secondary analysis. These treatments may have influenced lipid and glucose levels, partly explaining why we did not detect differences across racial and ethnic groups. Future studies should consider medication use when examining these biomarkers. Missing laboratory data may also have introduced bias, particularly for albumin and fasting glucose, because the reasons for missing values were not systematically documented and participants with available laboratory data may have differed from those without complete measurements.

Albumin was available for 50 of 92 participants and fasting glucose for 76 of 92 participants. Because the reasons for missing laboratory values were not systematically documented, we cannot exclude the possibility that participants with available laboratory data differed from those without complete measurements. This missingness may therefore have introduced selection bias and limited the representativeness of these estimates.

The results should also be viewed in the light of the study population. Participants were recruited from a single academic medical center, were well-educated, and volunteered for a nutrition-focused clinical trial; these characteristics may limit the generalizability of the results to a broader HF population. Trial participation may therefore have selected individuals who were more motivated or interested in nutrition and dietary change than the broader population of adults with HF. These characteristics may also have reduced the socioeconomic and behavioral heterogeneity needed to detect differences across racial and ethnic groups.

This analysis did not include sodium or selected micronutrients, despite their clinical relevance in HF. These measures were outside the scope of the present analysis, which was intentionally limited to a parsimonious set of energy and macronutrient measures given the small subgroup sizes and substantial missing dietary data. Their omission limits our ability to determine whether group differences may have existed in other clinically important aspects of dietary quality. Important social determinants of health, including food security, income, neighborhood food access and disadvantage, and health literacy, were not assessed in this analysis. These factors may influence both dietary intake and HF outcomes and could contribute to racial and ethnic differences that were not captured by the clinical and dietary measures examined here. Future research should include measures of these social and structural factors as well as dietary and clinical measures.

## 5. Conclusions

Among adults with chronic HF, type 2 DM, and overweight or obesity, no statistically significant racial or ethnic differences were identified in the dietary and laboratory measures available for analysis. However, the small subgroup sizes and substantial missing dietary data limit the precision and interpretation of these findings, and smaller or clinically meaningful differences cannot be excluded. These results should therefore be considered exploratory and should not be interpreted as evidence of equivalence across racial and ethnic groups. Larger studies with more complete dietary data are needed to better characterize potential racial and ethnic differences in nutrition and metabolic health in this population.

## Figures and Tables

**Table 1:** Baseline demographic and clinical characteristics by racial and ethnic group.

Characteristic	Overall (N = 92)	Black (*n* = 12)	White (*n* = 49)	Asian/Other (*n* = 17)	Hispanic (*n* = 14)	Omnibus *p*	η^2^ (95% CI)
Demographic Characteristics							
Male, *n* (%)	64 (69.6)	8 (66.7)	33 (67.3)	14 (82.4)	9 (64.3)	0.646 *	—
Married, *n* (%)	55 (64.0)	7 (58.3)	31 (63.3)	8 (47.1)	9 (64.3)	0.435 *	—
College education or higher, *n* (%)	71 (77.2)	8 (66.7)	37 (75.5)	16 (94.1)	10 (71.4)	0.130 *	—
Clinical Characteristics							
NYHA Class II, *n* (%)	69 (75.0)	9 (75.0)	34 (69.3)	14 (82.4)	12 (85.7)	0.781 *	—
Type 2 diabetes, *n* (%)	92 (100)	12 (100)	49 (100)	17 (100)	14 (100)	—	—
Age, years	58.5 ± 9.7	56.0 ± 6.5	60.3 ± 9.9	59.7 ± 7.8	52.6 ± 11.5	0.046	0.086 (0.000–0.188)
Left ventricular ejection fraction, %	38.7 ± 12.7	35.8 ± 14.0	40.0 ± 12.1	38.5 ± 13.7	37.1 ± 13.4	0.724	0.015 (0.000–0.063)
Body mass index, kg/m^2^	37.2 ± 6.3	36.2 ± 5.7	37.5 ± 6.3	35.4 ± 5.0	38.7 ± 8.1	0.470	0.028 (0.000–0.095)
Waist circumference, cm	120.7 ± 14.0	120.7 ± 13.1	121.5 ± 13.3	118.0 ± 15.0	121.1 ± 17.0 (14)	0.856	0.009 (0.000–0.043)

Footnote: Abbreviation: New York Heart Association (NYHA). Values are mean ± standard deviation unless otherwise indicated. Continuous variables were compared using one-way analysis of variance. * Fisher’s exact test was used for categorical variables when appropriate. η^2^ = eta squared.

## Data Availability

The article includes the original contributions presented in the study; further inquiries may be directed to the corresponding author.

## Abbreviations

The following abbreviations are used in this manuscript:

ANOVA	Analysis of Variance
DM	Diabetes Mellitus
HF	Heart Failure
NYHA	New York Heart Association
